# Isolation and preliminary screening of lactic acid bacteria for antimicrobial potential from raw milk

**DOI:** 10.3389/fmicb.2025.1565016

**Published:** 2025-03-05

**Authors:** Kawthar El Ahmadi, Khadija Haboubi, Hasnae El Allaoui, Yahya El Hammoudani, Mohamed Bouhrim, Bruno Eto, Abdelaaty A. Shahat, Rashed N. Herqash

**Affiliations:** ^1^Laboratory of Engineering Sciences and Applications, National School of Applied Sciences of Al Hoceima, Abdelmalek Essâadi University, Al-Hoceima, Morocco; ^2^Biological Engineering Laboratory, Faculty of Sciences and Techniques, Sultan Moulay Slimane University, Beni Mellal, Morocco; ^3^Laboratoires TBC, UFR3S, Département de Pharmacie, Université de Lille, Lille, France; ^4^Department of Pharmacognosy, College of Pharmacy, King Saud University, Riyadh, Saudi Arabia

**Keywords:** lactic acid bacteria, bacteriocins, antimicrobial activity, raw milk, food safety, pathogen inhibition, fermented foods, food microbiology

## Abstract

Lactic acid bacteria (LAB) are widely recognized for their role in food preservation and their potential to produce bacteriocins, natural antimicrobial peptides effective against a broad spectrum of foodborne pathogens. This study focuses on the isolation and characterization of bacteriocin-producing LAB strains from raw milk samples collected in southern and Northern Morocco. Phenotypic and genotypic methods were used to identify the isolated strains, and their antimicrobial activity was evaluated against common foodborne pathogens, including *Escherichia coli* and *Salmonella* spp. The results revealed several LAB strains with significant bacteriocin production and strong inhibitory effects against the target pathogens. These findings highlight the potential applications of these strains in the food industry, particularly for enhancing the safety and shelf life of fermented food products. This study provides a foundation for future research on the biotechnological exploitation of LAB as natural food preservatives.

## 1 Introduction

Lactic acid bacteria (LAB) are extensively studied microorganisms in the food industry due to their critical role in fermentation processes. They are widely utilized in the production of fermented foods, including dairy products, vegetables, and meat-based products. Beyond their contribution to texture and flavor development (Zapaśnik et al., 2022), LAB significantly enhances food safety by producing organic acids, such as lactic and acetic acids, which reduce the pH of the medium, thereby inhibiting the growth of pathogenic microorganisms.

Recent advancements in molecular biology techniques have unveiled a remarkable genomic diversity among LAB, leading to their classification into several genera, including Carnobacterium, Enterococcus, Lactobacillus, Lactococcus, Leuconostoc, Oenococcus, Pediococcus, and Weissella (El Ahmadi et al., 2024; Gemechu, 2015). These advancements have not only enriched our understanding of LAB but also highlighted their ability to produce bioactive compounds, such as bacteriocins ribosomally synthesized antimicrobial peptides effective against a broad spectrum of spoilage and pathogenic microorganisms (Abiola et al., 2022).

Despite the availability of various modern preservation techniques, including refrigeration, freezing, and sterilization, food spoilage and contamination remain major challenges in the food industry (El Allaoui et al., 2024; Widyastuti and Febrisiantosa, 2014). Additionally, consumer preferences are increasingly shifting toward natural and minimally processed foods, leading to a growing demand for alternative preservation methods that maintain food safety while preserving the product’s nutritional and sensory qualities (El Hammoudani et al., 2024). In response to these trends, LAB and their metabolites, particularly bacteriocins, have emerged as promising candidates for natural preservation strategies (Ayivi et al., 2020).

Bacteriocins are characterized by their stability, effectiveness against antibiotic-resistant pathogens, and specificity, making them ideal for application in the food industry. Their ability to act as natural preservatives aligns with the demand for safer and more sustainable food production practices (Allaoui et al., 2024; Beshkova and Frengova, 2012). As a result, the isolation and characterization of LAB strains capable of producing bacteriocins have garnered significant attention as a potential solution to enhance food safety and quality (Daly and Davis, 1998).

The present study aims to isolate and characterize bacteriocin-producing LAB strains from raw milk collected from Southern and Northern Morocco. The antimicrobial activity of these isolates is evaluated against foodborne pathogens such as *Escherichia coli* and *Salmonella* spp. The results of this study are expected to contribute to the development of natural food preservation systems and further explore the industrial applications of LAB and their bioactive metabolites.

## 2 Materials and methods

### 2.1 Sampling and milk collection techniques

Raw milk samples were collected aseptically from 11 cows on farms in southern (9) and northern (2) Morocco. Only clinically healthy cows, without antibiotic treatment in the previous 3 months, were included to avoid interference with the isolation of lactic acid bacteria (LAB). Selection criteria included parity (cows with at least one completed lactation), milk production (high >15 L/day, moderate 5–15 L/day), breed (Holstein-Friesian, Montbeliarde and local breeds) and lactation stage (mid-lactation, 2ᵉ–6ᵉ months postpartum). Approximately 100 mL of milk was collected after removal of first sprays to avoid contamination. Samples were stored at 4°C and transported to the laboratory within 4 h, with detailed labeling to ensure traceability.

### 2.2 Enumeration of lactic acid bacteria

The enumeration of lactic acid bacteria was carried out using MRS (Man-Rogosa-Sharpe) medium and nutrient agar, which are commonly used for the growth and enumeration of lactic acid bacteria and total microbial flora, respectively (Table 1). The decimal dilution method was employed to prepare serial dilutions of the samples, ensuring accurate quantification of the bacterial populations. For lactic flora, incubation was conducted under anaerobic conditions at 37°C for 48 h to promote the growth of lactic acid bacteria (Vinderola and Reinheimer, 2000).

**Table 1:** Media and incubation conditions for enumeration of lactic acid bacteria.

Microorganism	Isolation medium	Incubation temperature (°C)	Duration (hours)	Environment
Lactic acid bacteria (LAB)	M17	37	48 h	Anaerobic
	MRS	37	48 h	Anaerobic

### 2.3 Isolation and purification of LAB strains

#### 2.3.1 Isolation

**Figure 1:**
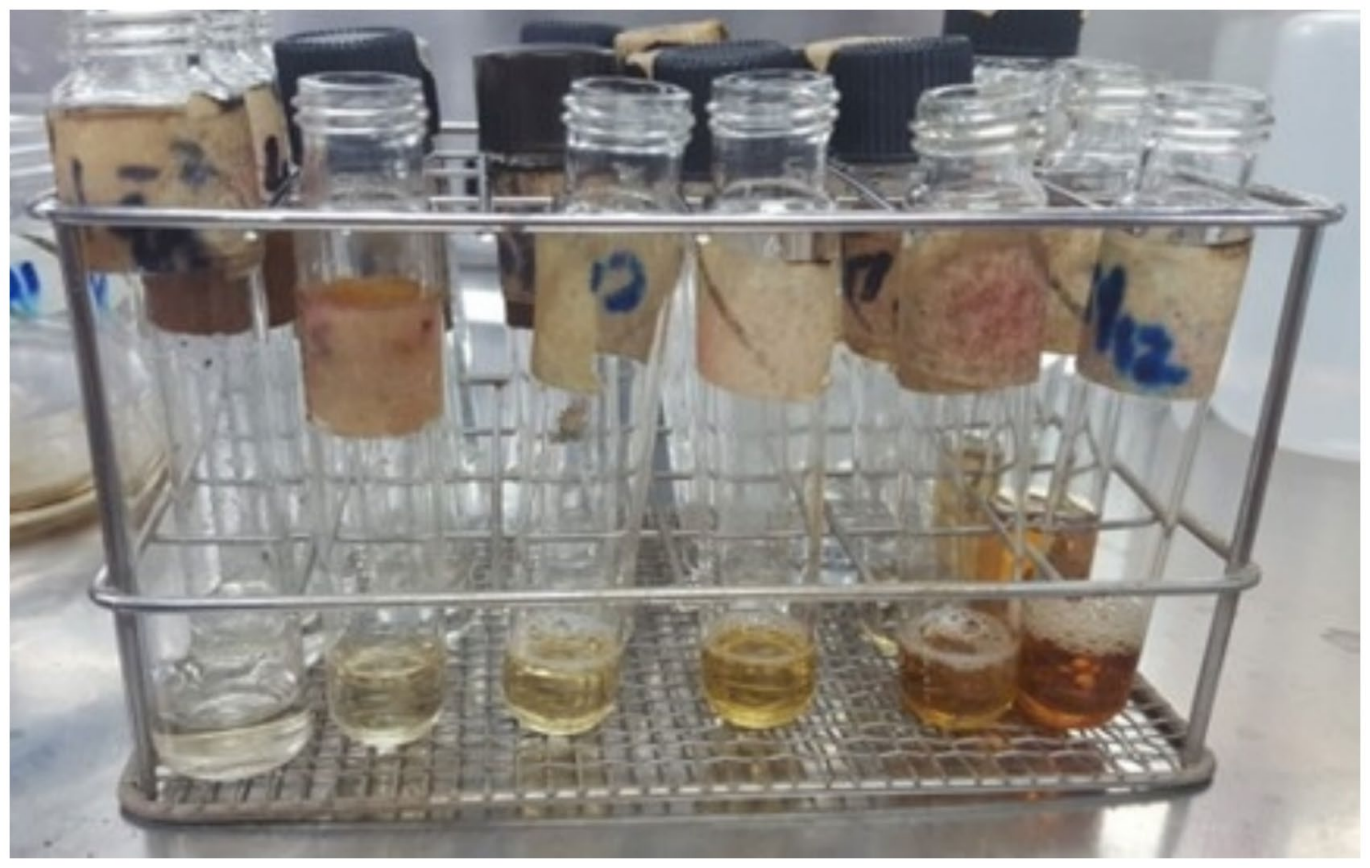
Fermentation tubes for lactic acid bacteria cultivation.

The isolation of lactic acid bacteria (LAB) strains was conducted using raw cow milk samples collected from Southen and northern Morocco. Initially, 10 grams of milk were mixed with 90 mL of a sterile saline solution (1% NaCl) to create a 10% suspension. Decimal dilutions ranging from 10^−1^ to 10^−10^. were prepared to reduce microbial concentration, and 0.1 mL of each dilution was plated on solidified MRS (Man, Rogosa, Sharpe) or M17 agar media (Figure 1). These plates were incubated anaerobically at 37°C for 24–48 h. Following incubation, colonies with distinct morphological characteristics, such as size, shape, and color, were selected for further analysis (Batdorj et al., 2006).

#### 2.3.2 Purification

The purification of LAB strains involved successive streaking of the selected colonies onto fresh MRS or M17 agar plates to ensure the isolation of pure colonies. This process was repeated until uniform colony morphology was achieved. Pure colonies were then transferred to MRS or M17 broth and incubated at 37°C for 24 h to confirm purity and viability. Additional streaking on agar plates was performed to verify the consistency of the cultures. For short-term storage, purified strains were maintained on MRS slants at 4°C, with re-streaking required every 2 weeks. For long-term preservation, the strains were stored in a glycerol-based medium containing 70% skim milk and 0.05% yeast extract, kept at −80°C to maintain viability. This systematic approach ensured reliable isolation and purification of LAB strains for subsequent analyses (Abdullahi, 2019).

### 2.4 Identification of LAB strains

#### 2.4.1 Cultural and morphological characteristics

Macroscopic observations were conducted on MRS and M17 agar plates to evaluate colony characteristics such as size, shape, and color. For microscopic examination, bacterial smears were prepared from the isolated colonies and subjected to Gram staining. This method was used to identify the Gram reaction and cellular morphology of the isolates, aiding in their classification as Gram-positive bacteria. These analyses provided essential information for the identification of LAB strains (Veselá et al., 2019).

#### 2.4.2 Physiological characteristics

The physiological characteristics of the isolates were assessed by evaluating their growth under varying environmental conditions. Temperature tolerance was tested by incubating the isolates at 10°C, 40°C, and 45°C for a duration of 7 days. Additionally, salt tolerance was determined by monitoring growth in media supplemented with NaCl at concentrations of 2, 4, and 6.5%, also over a 7-day incubation period. These tests provided insights into the adaptability of the isolates to different conditions (Bhardwaj et al., 2012).

#### 2.4.3 Biochemical characteristics

2H_2_O_2_ 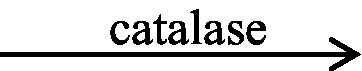 2H_2_O + O_2_

The biochemical characteristics of the isolates were evaluated using the catalase test. A single bacterial colony was emulsified in a drop of hydrogen peroxide (H₂O₂) on a clean glass slide. The test was conducted to detect the presence of the catalase enzyme, which breaks down hydrogen peroxide into water and oxygen. The absence of gas bubble formation indicated a negative catalase reaction, a typical feature of lactic acid bacteria (LAB) (Khedid et al., 2009).

### 2.5 Conservation of LAB strains

LAB strains were preserved using both short-term and long-term storage methods. For short-term storage, the strains were maintained on inclined MRS agar slants at 4°C, with subculturing performed every 2 weeks to ensure their viability. For long-term preservation, the strains were stored in a cryoprotective medium composed of 70% skimmed milk enriched with 0.05% yeast extract and 0.05% glucose, combined with 50% glycerol, and kept at −80°C to maintain their stability over extended periods (Amaral et al., 2020).

### 2.6 Evaluation of acidifying activity

#### 2.6.1 Acidification in skimmed milk

The acidifying activity of LAB strains was assessed by inoculating 10 mL of sterilized skimmed milk with 1% of the LAB culture. The samples were incubated at 37°C for 24 h. Coagulation was monitored between 18 and 24 h to evaluate acid production. The acidity of the milk was quantified using a titration method with standardized NaOH, and the results were expressed in Dornic degrees (°D) (Cavanagh et al., 2015).

#### 2.6.2 Acidification kinetics

The acidification kinetics of highly acidifying LAB strains were evaluated by measuring the pH and acidity of the culture medium at 2-h intervals during the incubation period. This allowed for the assessment of the rate and extent of acid production over time (Ruvalcaba-Gómez et al., 2022).

### 2.7 Antimicrobial activity and confirmation of bacteriocin production

#### 2.7.1 Reference strains

The antimicrobial activity of LAB was tested against *E. coli* and *Salmonella* spp. (Table 2).

**Table 2:** Reference strains used.

Strain	GRAM	Respiration type	Reference strain
*E. coli*	–	Aerobic	ATCC 25922
*Salmonella*	–	Aerobic	ATCC 14028

#### 2.7.2 Agar well diffusion method

**Figure 2:**
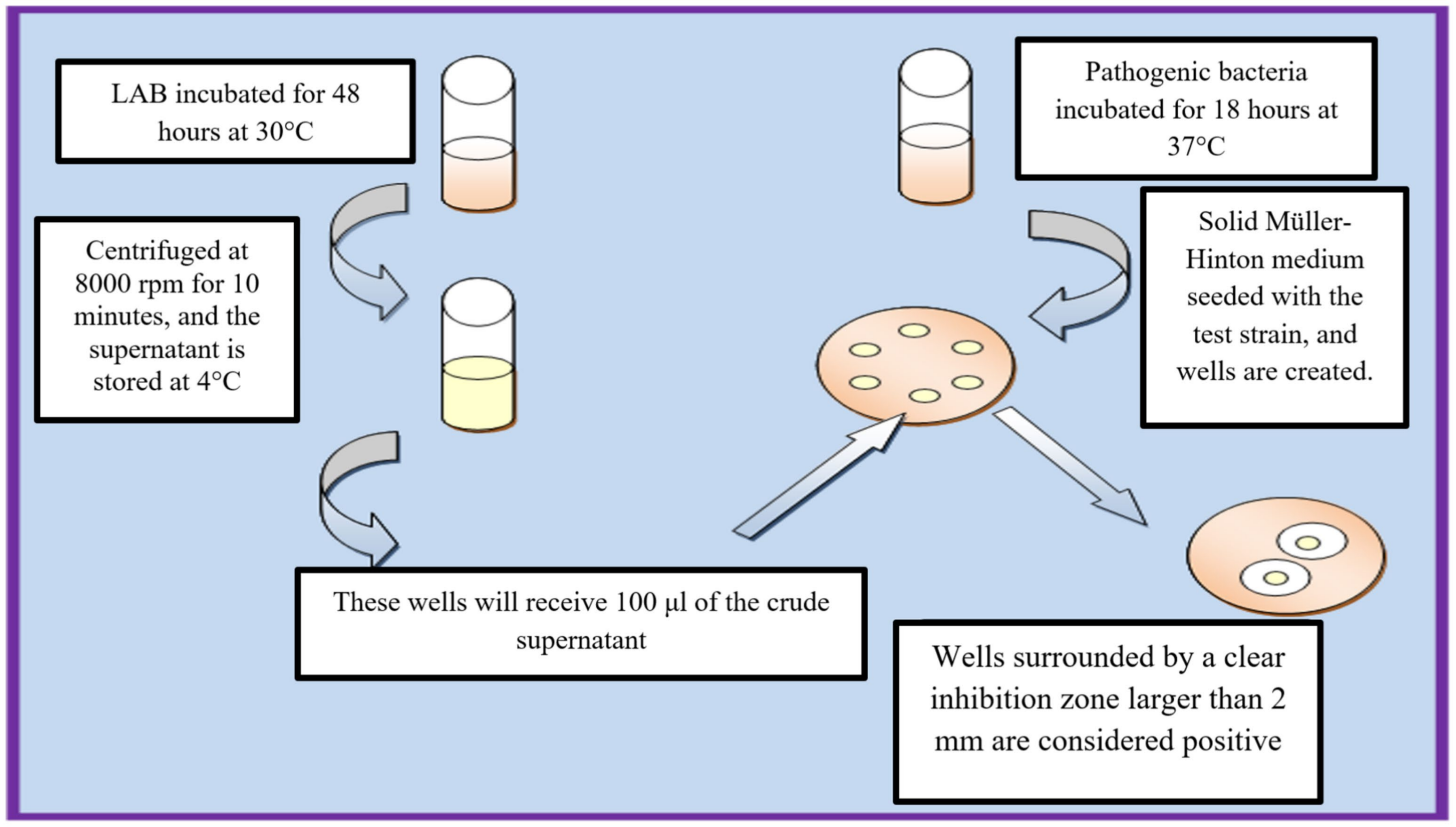
Workflow of agar well diffusion method for bacteriocin activity assessment.

The production of bacteriocins by LAB strains was evaluated using the agar well diffusion method. LAB strains were cultivated in liquid MRS medium and incubated at 37°C for 48 h under anaerobic conditions (Figure 2). The cultures were then centrifuged at 8,000 rpm for 10 min at 4°C to obtain a cell-free supernatant (CFS), which was subsequently filtered through a 0.22 μm membrane filter to remove residual bacterial cells. To eliminate the potential inhibitory effect of organic acids, particularly lactic acid, the pH of the CFS was adjusted to 7.0 using sterile 1 N NaOH. This step ensured that any observed antimicrobial activity was due to bacteriocins rather than pH-induced pathogen inhibition. A control experiment was also performed using non-neutralized supernatant to compare inhibition zones. For the antimicrobial activity assay, Mueller-Hinton agar plates seeded with *Escherichia coli* and *Salmonella* spp. were prepared, and wells were created using a sterile punch. Each well was filled with 100 μL of the neutralized LAB supernatant, and the plates were incubated at 30°C for 24 h. The antimicrobial activity was determined by measuring the zones of inhibition, with a diameter of ≥2 mm indicating positive activity (Lewus and Montville, 1991).

#### 2.7.3 Confirmation of bacteriocin production

To confirm that the antimicrobial activity was due to bacteriocins rather than other antimicrobial compounds (such as organic acids or hydrogen peroxide), the following tests were conducted:

##### 2.7.3.1 pH neutralization test (control for organic acids)

The pH of the LAB supernatant was adjusted to 7.0 using NaOH (1 N) before performing the antimicrobial activity test. The agar well diffusion assay was then repeated to determine whether the inhibition zones persisted.

##### 2.7.3.2 Catalase treatment (control for hydrogen peroxide)

The cell-free supernatant was treated with catalase (100 μg/mL) and incubated at 37°C for 30 min to degrade hydrogen peroxide. The antimicrobial test was repeated to assess any reduction in inhibition zones.

##### 2.7.3.3 Protease sensitivity test (confirmation of the proteinaceous nature of bacteriocins)

The supernatant was treated separately with trypsin (1 mg/mL) and proteinase K (0.1 mg/mL) at 37°C for 1 h. The disappearance of inhibition zones after enzymatic treatment confirmed that the antimicrobial activity was due to proteinaceous compounds, specifically bacteriocins.

## 3 Results

### 3.1 Isolation

**Figure 3:**
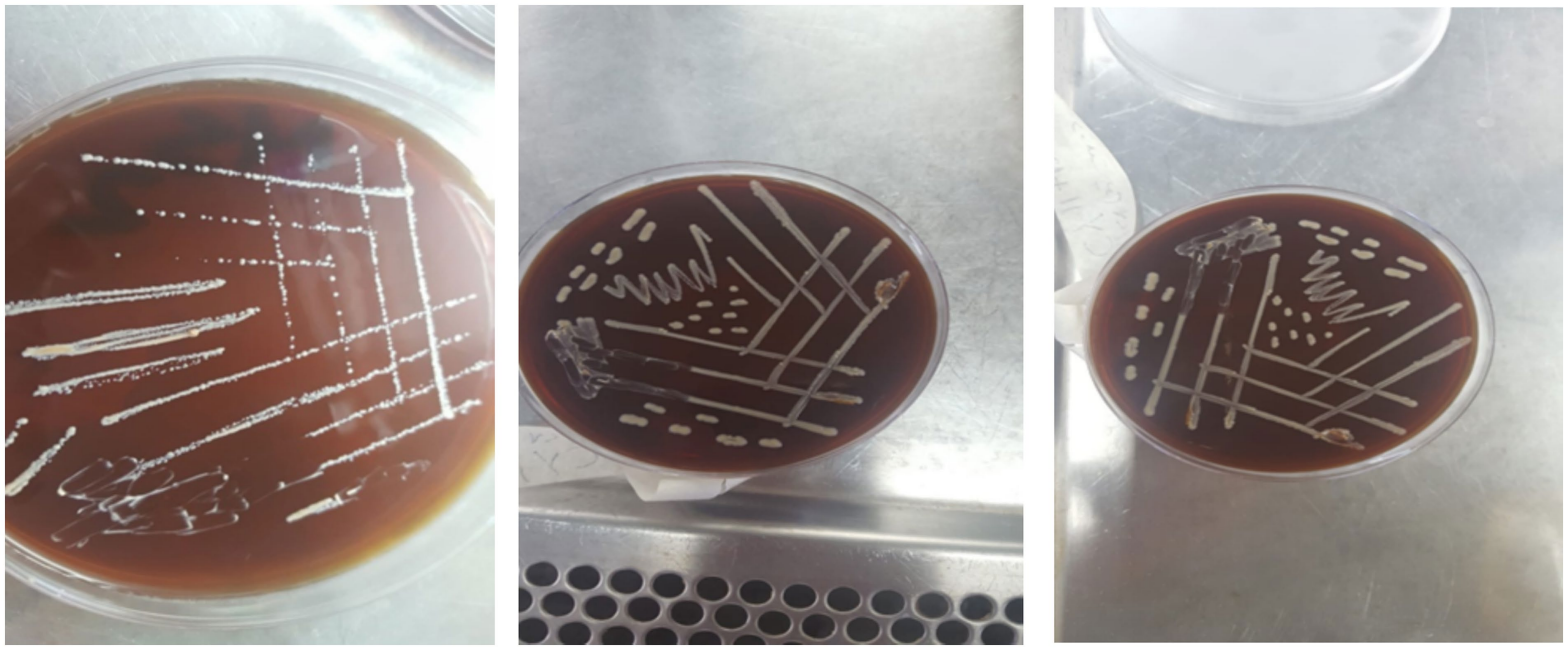
Growth patterns of isolated lactic acid bacteria on solid media.

Twenty isolated colonies varied in size, exhibited a circular shape with regular, irregular, or eroded edges, and displayed colors ranging from white to transparent or grayish-white (see Figure 3). The colonies underwent purification and phenotypic identification using Gram staining and microscopy to distinguish cocci from rod-shaped LAB. Biochemical tests (catalase activity, gas production, and carbohydrate fermentation) classified the isolates into genera. 16S rRNA sequencing confirmed taxonomic classification, identifying five genera: Lactobacillus, Lactococcus, Leuconostoc, Pediococcus, and Streptococcus.

### 3.2 Identification

#### 3.2.1 Distribution of lactic acid bacteria

**Figure 4:**
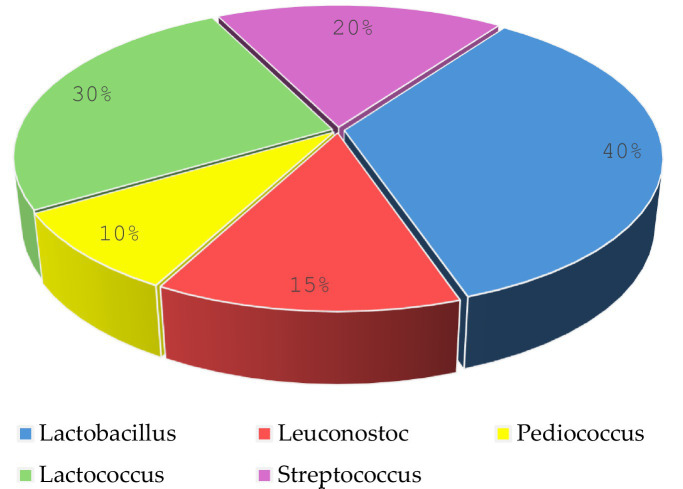
Distribution of lactic acid bacteria isolated from cow’s milk.

Microscopic observation revealed two cell morphologies: cocci and rods. Cocci (diplococci, chain-forming cocci, or tetrads) accounted for 59% of the total isolates and were represented by the genera Lactococcus, Leuconostoc, Pediococcus, and Streptococcus. The rod-shaped cells observed were classified under the genus Lactobacillus, representing 41% of the total isolates (Figure 4).

#### 3.2.2 Morphological study

**Figure 5:**
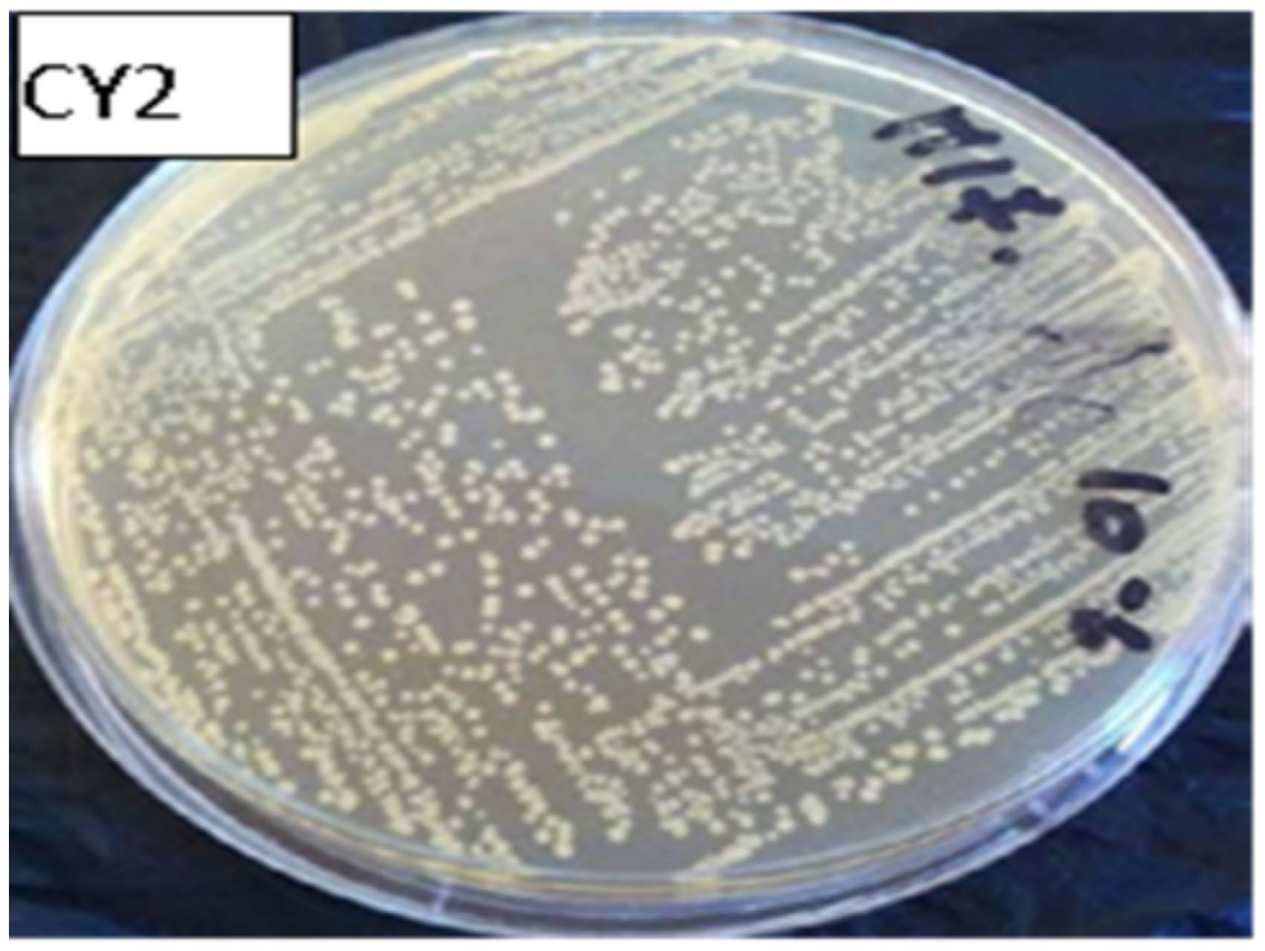
Macroscopic observation of bacterial colonies on M17 agar.

**Figure 6:**
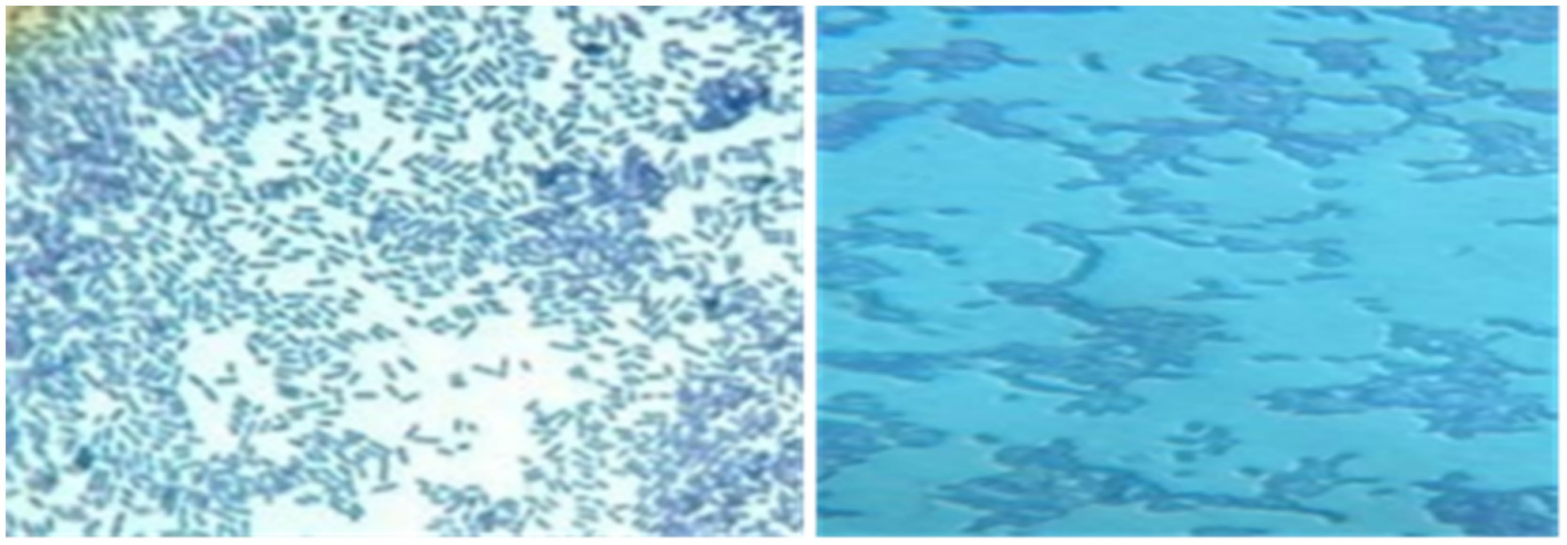
Microscopic observation after gram staining at 100x magnification of rods and cocci.

Macroscopic examination on MRS or M17 agar revealed colonies of varying sizes, with whitish or cream coloration and a circular shape with regular edges (Figure 5). The colony diameter ranged from 0.1 to 0.5 mm (Figure 6).

**Figure 7:**
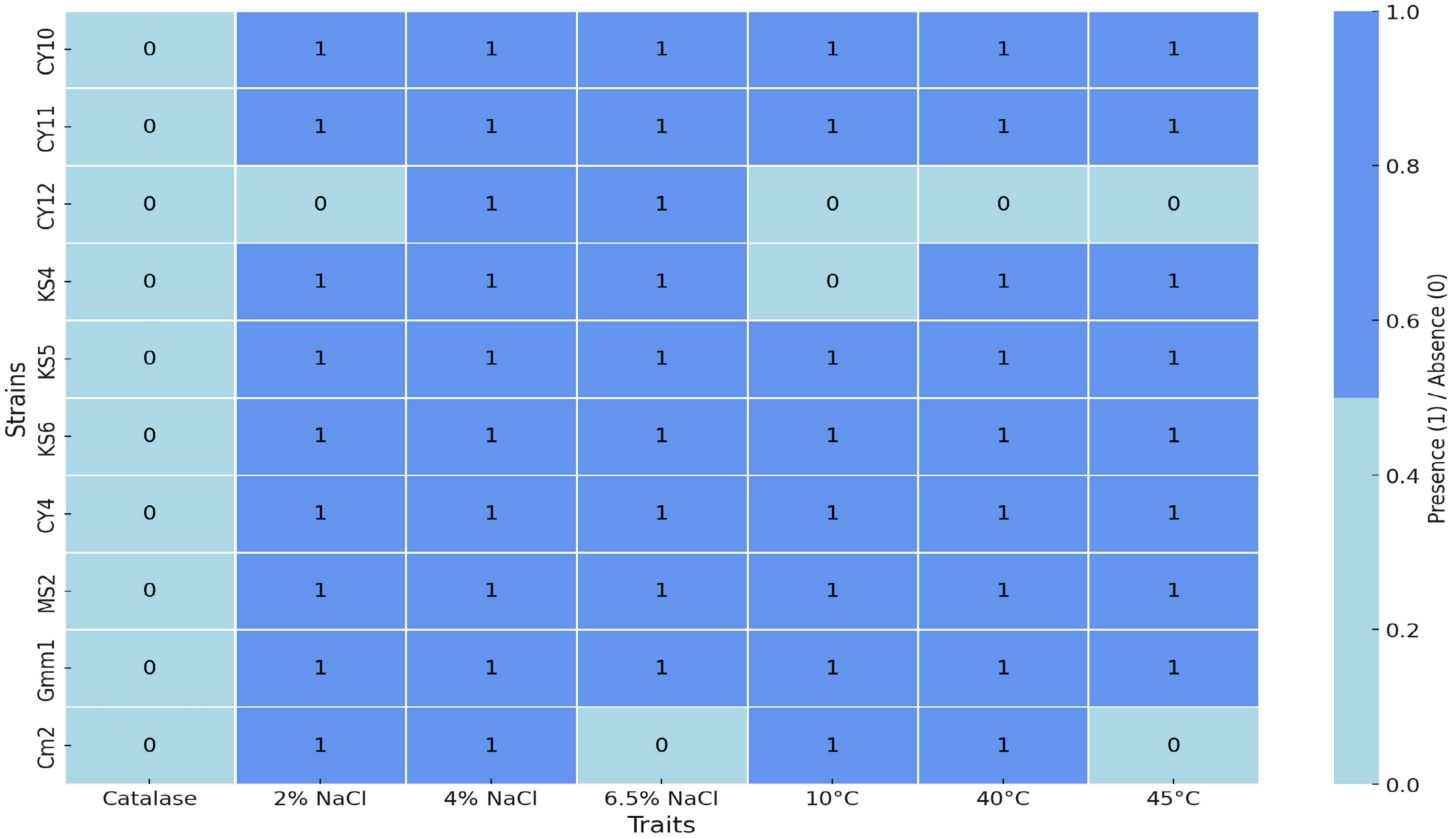
Heatmap of physiological and biochemical traits.

After Gram staining, microscopic observations confirmed that the isolated lactic acid bacteria were Gram-positive, with two main cell morphologies identified: cocci and rods. The cocci were arranged in clusters, chains, or pairs, while the rods were observed in chains or pairs. Strains such as CY1, CY2, CY3, CY5, CY6, CY8, MC1, MC2, MC3, MC4, MC5, MC6, KS5, and KS7 were classified as cocci. Meanwhile, strains CY4, CY7, CY9, CY10, CY11, CY12, KS1, KS2, KS4, and KS6 were identified as Gram-positive rods. These rod-shaped strains were also catalase-negative, a typical characteristic of lactic acid bacteria. The specific modes of association, including diplococci, chains, or isolated forms, varied between the strains and are detailed in Figure 7. These observations provided an initial basis for classifying the isolates according to their Gram reaction, cellular morphology, and association patterns, supporting further phenotypic and genotypic identification efforts (Table 3).

**Table 3:** Morphological criteria of lactic acid bacteria isolated from cow’s milk.

Strains	Gram	Catalase	Shape	Mode of association
CY1	+	−	Cocci	In chains
CY2	+	−	Cocci	In chains
CY3	+	−	Cocci	In chains
CY4	+	−	Rod	In chains
CY5	+	−	Cocci	Diplococci
CY6	+	−	Cocci	Isolated
CY7	+	−	Rod	In chains
CY8	+	−	Cocci	In chains
CY9	+	−	Rod	In chains
CY10	+	−	Rod	In chains
CY11	+	−	Rod	Diplococci, isolated
CY12	+	−	Rod	Diplococci, isolated
MC1	+	−	Cocci	Isolated
MC2	+	−	Cocci	Diplococci, isolated
MC3	+	−	Cocci	Diplococci, isolated
MC4	+	−	Cocci	In chains
MC5	+	−	Cocci	Diplococci
MC6	+	−	Cocci	Diplococci, isolated
KS1	+	−	Rod	In chains
KS2	+	−	Rod	In chains
KS4	+	−	Rod	In chains
KS5	+	−	Cocci	In chains
KS6	+	−	Rod	In chains
KS7	+	−	Cocci	Diplococci, isolated

### 3.3 Physiological and biochemical traits of lactic acid bacteria

#### 3.3.1 Growth in saline medium

The heatmap in Figure 7 illustrates the physiological and biochemical traits of the tested strains, with light blue indicating the absence of a trait (value 0) and cornflower blue representing its presence (value 1). The ability of the isolates to grow under hyper-saline conditions (6.5% NaCl) and varying temperatures was assessed to evaluate their physiological robustness and adaptability. The results, represented in Figure 7, revealed that strains CY10, CY11, CY12, KS4, KS5, KS6, CY4, MS2, and GMM1 demonstrated tolerance to high salinity, thriving in media containing 6.5% NaCl. This resilience under osmotic stress highlights their potential application in fermentation processes that often require high salt concentrations, such as in the production of certain cheeses and fermented vegetables.

#### 3.3.2 Growth at different temperatures

**Figure 8:**
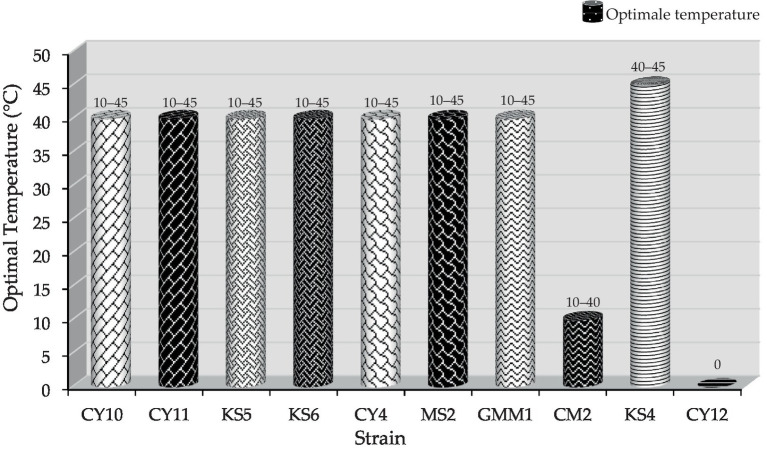
Optimal temperature and growth range of different strains.

In addition to salinity tolerance, the isolates were evaluated for their temperature adaptability. Eight strains (CY10, CY11, KS5, KS6, CY4, MS2, GMM1, and CM2) displayed the ability to grow at multiple temperatures, with optimal growth observed for specific strains at 10, 40, or 45°C, as illustrated in Figure 8. This highlights their adaptability and potential for applications in diverse environmental and industrial conditions. Conversely, certain strains exhibited more restricted growth patterns. Strain CY12 failed to grow at any of the tested temperatures (10, 40, or 45°C), indicating limited adaptability. Strain KS4 displayed growth only at 40 and 45°C, suggesting a preference for higher temperatures. Similarly, strain CM2 exhibited selective growth at 10 and 40°C, highlighting its temperature-specific tolerance. These results, visually represented in Figure 8, provide insights into the physiological adaptability of the strains, offering valuable information for their potential industrial applications.

#### 3.3.3 Catalase test

The catalase test results revealed that all the isolated strains were catalase-negative, a typical characteristic of lactic acid bacteria. This biochemical trait underscores the strains’ relevance for applications in anaerobic fermentation processes where oxygen is limited.

### 3.4 Study of technological characteristics

#### 3.4.1 Study of the acidification capacity of strains isolated from cow’s milk

**Figure 9:**
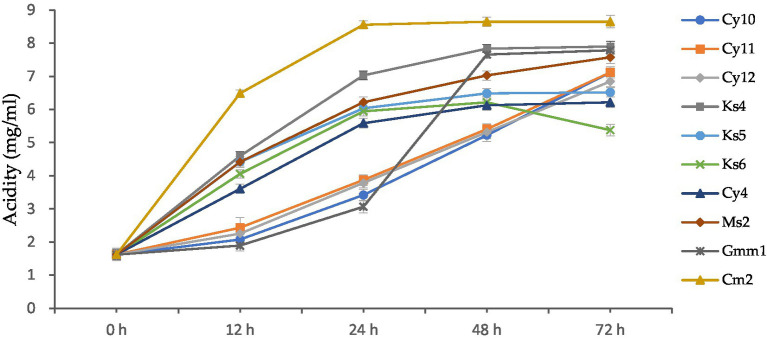
Kinetics of acid production (mg/mL) by lactic acid bacteria strains at different incubation periods (0–72 h).

Acidifying activity is one of the main functions of lactic acid bacteria. The results of the monitoring of the acidifying activity of strains isolated from cow’s milk are presented in the Figure 9. The figure illustrates the evolution of acidity (in mg/ml) in lactic acid bacteria strains over a 72-h incubation period. Initially, at 0 h, all strains exhibit the same acidity level of 1.621 mg/mL, indicating a uniform starting point for all cultures.

After 12 h, variations in acidity levels begin to emerge. For instance, CY10 reaches an acidity of 2.071 mg/mL, while CY11 and CY12 increase to 2.432 mg/mL and 2.252 mg/mL, respectively. At this stage, GMM1 shows the lowest increase, with an acidity of 1.891 mg/mL, suggesting slower metabolic activity compared to the other strains. At 24 h, the acidity continues to increase significantly. Strains like KS4 and CM2 stand out with notable values of 7.026 mg/mL and 6.485 mg/mL, reflecting their strong acid production activity. In contrast, GMM1 remains behind, with an acidity of 3.062 mg/mL, highlighting its limited growth compared to the others. By 48 h, some strains maintain a rapid increase in acidity. KS4 remains the leader, reaching an acidity of 7.836 mg/mL, the highest among all strains at this point. Meanwhile, other strains, such as KS6 and CY4, show more moderate increases, reaching 6.215 and 6.125 mg/mL, respectively. This suggests differences in metabolic capacities or physiological adaptations to the growth medium. Finally, at 72 h, the results reveal significant differences between the strains. CM2 exhibits the highest acidity, reaching 8.647 mg/mL, followed by KS4 with 7.896 mg/mL, suggesting that these two strains have the greatest biotechnological potential in terms of acid production. On the other hand, KS6 shows a decrease in acidity to 5.375 mg/mL, which might indicate metabolic limitations or nutrient depletion. GMM1, although steadily increasing, reaches 7.785 mg/mL, confirming its lower activity compared to strains like CM2 and KS4.

The acidity (in mg/ml) was calculated by titration using a standardized method. The lactic acid produced during fermentation reacts with a titrant, and the volume of titrant required to neutralize the acid is converted into milligrams of lactic acid per milliliter (mg/ml) based on a known equivalence factor.

These findings highlight the variability in the metabolic capabilities of the studied lactic acid bacteria strains. The observed differences could be exploited to select strains suitable for specific biotechnological applications, such as the production of fermented dairy products or other industrial fermentation processes.

### 3.5 Antimicrobial activity and confirmation of bacteriocin production

#### 3.5.1 Antagonistic activity of strains against *Escherichia coli*

The antagonistic activity of lactic acid bacteria (LAB) strains against *Escherichia coli* was evaluated by measuring the inhibition zone diameters (Table 4). Statistical analysis using one-way ANOVA followed by Tukey’s *post hoc* test revealed significant differences (*p* < 0.05) among the strains. CY10, CY11, CY12, KS6, CY4, MS2, MOY1, and CM2 exhibited inhibition zones ranging from 9 to 18 mm. Notably, inhibition was considered positive for diameters exceeding 2 mm. Among the tested strains, CY11, MS2, and MOY1 showed the highest inhibition, suggesting strong bacteriocin production or similar antimicrobial activity.

**Table 4:** Antagonistic activity of LAB strains against *E. coli*.

Strain	Diameter of zone of inhibition (mm)
CY10	12.167 ± 0.3511^bc^
CY11	18.033 ± 0.1527^e^
CY12	13.1 ± 0.30^c^
KS5	9.033 ± 0.3055 ^b^
KS6	14.967 ± 0.208^d^
CY4	15.0 ± 0.10 ^d^
MS2	17.0 ± 0.10^ed^
MOY1	18.067 ± 0.153^e^
CM2	9.0 ± 0.10 ^b^
KS4	0.0 ± 0.00^a^

The diameter of the zone of inhibition is expressed as mean ± standard deviation. Different letters (a, b) in each column indicate statistically significant differences at *p* < 0.05.

**Figure 10:**
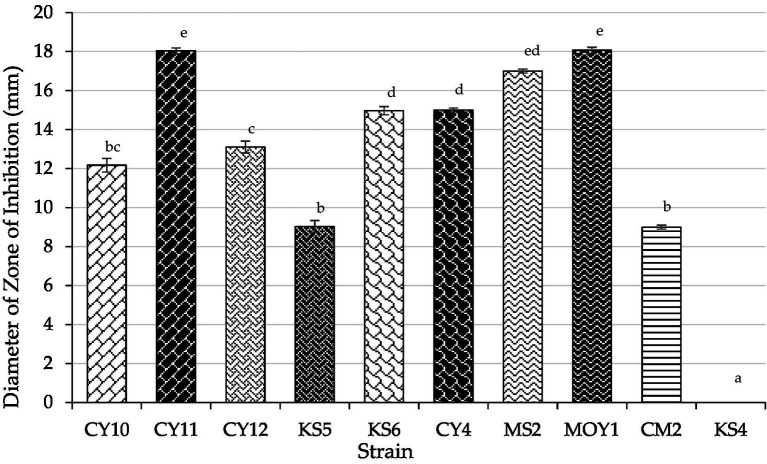
Inhibition zones of LAB strains against *E. coli* (Mean ± SD, ANOVA results).

In contrast, KS4 exhibited no inhibition, indicating lower antagonistic potential. The results, illustrated in Figure 10, highlight the potential of these strains for antimicrobial applications, particularly in combating *E. coli*. The observed variation in inhibition diameters, confirmed by statistical significance, reflects the diversity of bacteriocin production and antimicrobial effectiveness among the strains.

Each value represents the mean of three replicates, and the bars represent the standard error. Different letters in each column indicate statistically significant differences at *p* < 0.05 according to one-way ANOVA followed by Tukey’s *post hoc* test.

#### 3.5.2 Antagonistic activity of strains against *Salmonella*

Similarly, the antagonistic activity of LAB strains against *Salmonella* was analyzed through inhibition zone measurements and subjected to one-way ANOVA and Tukey’s *post-hoc* test (Table 5). The results showed significant variations (*p* < 0.05) among the tested strains, with CY10, CY11, CY12, KS6, MS2, and MOY1 exhibiting inhibition zones ranging from 10 mm to 23 mm. CY10 and KS6 showed the largest inhibition zones, indicating strong antagonistic effects, while KS4 exhibited no inhibition, suggesting lower antimicrobial efficacy.

**Table 5:** Antagonistic activity of LAB strains against *Salmonella*.

Strain	Diameter of zone of Inhibition (mm)
CY10	23.3 ± 0.435^a^
CY11	15.033 ± 0.208^d^
CY12	18.066 ± 0.378^f^
KS5	0.0 ± 0.00^a^
KS6	17.066 ± 0.152^e^
CY4	0.0 ± 0.00^a^
MS2	15.3 ± 0.360^d^
MOY1	13.166 ± 0.251^c^
CM2	0.0 ± 0.00^a^
KS4	15.166 ± 0.450^d^

The diameter of the zone of inhibition is expressed as mean ± standard deviation. Different letters (a, b) in each column indicate statistically significant differences at *p* < 0.05.

**Figure 11:**
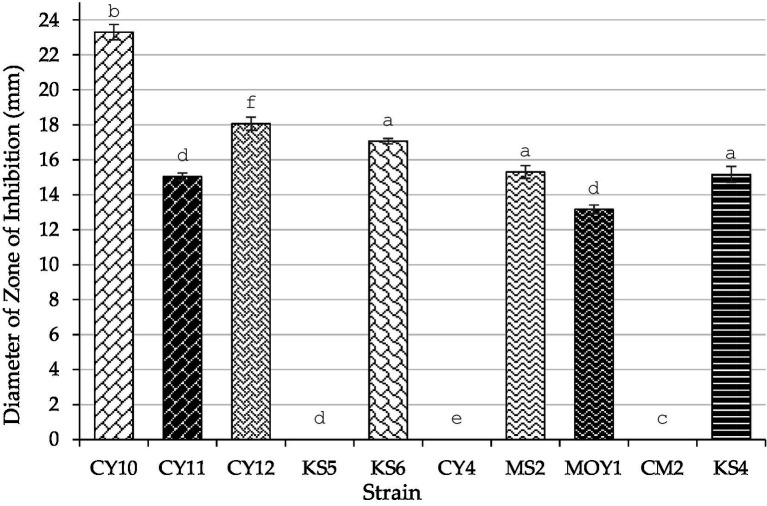
Inhibition zones of LAB strains against *Salmonella* (Mean ± SD, ANOVA results).

As shown in Figure 11, these findings emphasize the potential of LAB strains for biotechnological applications, particularly in food safety and pathogen control. The variability in inhibition diameters, statistically validated, underscores the functional diversity of these strains, which could be leveraged for targeted antimicrobial applications.

Each value represents the mean of three replicates, and the bars represent the standard error. Different letters in each column indicate statistically significant differences at *p* < 0.05 according to one-way ANOVA followed by Tukey’s *post hoc* test.

#### 3.5.3 Confirmation of bacteriocin activity

To confirm that the antimicrobial activity observed in the agar well diffusion assay was not due to other inhibitory compounds, such as organic acids or hydrogen peroxide, additional control experiments were performed. Based on preliminary antimicrobial screening, only the five most active strains (CY2, CY4, MC2, KS1, and KS2) were selected for further bacteriocin confirmation tests.

##### 3.5.3.1 Effect of pH neutralization

To rule out the contribution of organic acids, the supernatant of LAB cultures was adjusted to pH 7.0 using NaOH (1 N) before performing the antimicrobial assay. The inhibition zones remained unchanged, indicating that organic acids were not responsible for the observed activity.

##### 3.5.3.2 Effect of catalase treatment

Since LAB can produce hydrogen peroxide, which has antimicrobial effects, the supernatant was treated with catalase (100 μg/mL at 37°C for 30 min) before antimicrobial testing. No significant reduction in inhibition zones was observed, confirming that hydrogen peroxide was not a major factor in the antimicrobial effect.

##### 3.5.3.3 Effect of protease treatment

To determine whether the antimicrobial activity was due to proteinaceous compounds (bacteriocins), the supernatant was treated with trypsin (1 mg/mL) and proteinase K (0.1 mg/mL) at 37°C for 1 h before antimicrobial testing. The inhibition zones completely disappeared or were significantly reduced, confirming that the antimicrobial compounds were proteins, consistent with bacteriocins (Table 6).

**Table 6:** Effect of pH neutralization, catalase treatment, and protease treatment on antimicrobial activity.

Strain	Control (no treatment) (mm)	After pH neutralization (mm)	After catalase treatment (mm)	After protease treatment (mm)
CY2	15	14	15	0 (No inhibition)
CY4	13	12	13	1 (Minimal inhibition)
MC2	14	14	14	0 (No inhibition)
KS1	16	16	16	0 (No inhibition)
KS2	12	12	12	1 (Minimal inhibition)

## 4 Discussion

### 4.1 Comparison with previous studies on LAB and their bacteriocins

This study highlights the diversity of physiological, biochemical, and antimicrobial capabilities of lactic acid bacteria (LAB) isolated from raw cow’s milk, emphasizing their significant potential for industrial and biotechnological applications. The isolation process identified several LAB genera, including Lactobacillus, Streptococcus, Enterococcus, Lactococcus, and Pediococcus, each exhibiting specific characteristics that influence their technological utility.

The antimicrobial activity of LAB strains observed in this study aligns with findings from previous research on LAB-derived bacteriocins. The inhibition zones recorded against *Escherichia coli* and *Salmonella* spp. reached up to 23 mm, with strains CY11, MS2, and MOY1 exhibiting the strongest antimicrobial effects. These findings are consistent with earlier studies demonstrating that certain LAB strains produce bacteriocins capable of inhibiting a wide range of foodborne pathogens.

Several studies have also reported that bacteriocin activity is influenced by environmental factors such as pH, temperature, and salt concentration (Manoharan and Balasubramanian, 2022). While these parameters were not fully explored in this study, prior research has shown that some bacteriocins retain antimicrobial activity even under extreme conditions, reinforcing their potential for food preservation applications. Future studies should evaluate the stability of the bacteriocins produced by the identified strains under different pH levels, temperature ranges, and food matrices to assess their industrial applicability.

### 4.2 Study limitations

Despite the promising results, several limitations must be acknowledged to refine the interpretation of findings and guide future research. A primary limitation is the identification of LAB strains based solely on biochemical and phenotypic characteristics, which do not allow for precise species- or strain-level classification (Zou et al., 2018). To address this, future studies will incorporate 16S rRNA gene sequencing and whole-genome sequencing to confirm strain identity and uncover bacteriocin biosynthetic pathways. This will not only improve taxonomic classification but also provide insights into the genetic regulation of bacteriocin production, potentially enabling genetic modifications to enhance biosynthesis (Yang et al., 2012).

Another key limitation is the lack of bacteriocin purification and structural characterization. While this study confirmed that the antimicrobial activity is protein-based through pH neutralization and enzyme treatments, the specific bacteriocins responsible were not isolated or analyzed. Future research will employ chromatographic techniques (HPLC, size-exclusion chromatography) and mass spectrometry to purify and characterize these peptides, allowing for a deeper understanding of their structure, antimicrobial properties, and mechanisms of action. Given the rising concern of antimicrobial resistance (AMR), bacteriocins promise as alternatives to conventional antibiotics, particularly against drug-resistant pathogens such as MRSA and VRE. Certain well-characterized bacteriocins, such as nisin and pediocin, exert their effects through pore formation in bacterial membranes, enzymatic inhibition of cell wall biosynthesis, or intracellular targeting of essential metabolic pathways (Reis et al., 2012). Future studies should investigate whether the bacteriocins identified in this study share similar structural or functional properties, as this could support their development as therapeutic agents (Ahmadi et al., 2024; Kurnianto et al., 2024).

Beyond characterization, stability assessments are crucial for determining the feasibility of bacteriocin applications. This study did not evaluate their resistance to environmental factors such as temperature, pH, and oxidative stress, which are essential for their effectiveness in food preservation and medical use. Previous studies indicate that some bacteriocins lose activity under extreme conditions, limiting their industrial applicability. Future research will assess bacteriocin stability across a temperature range of 4 to 60°C, pH variations, and oxidative conditions, ensuring their robustness for both food safety and pharmaceutical applications. Additionally, their antimicrobial efficacy will be tested in real food matrices, such as dairy, meats, and fermented beverages, to validate their role as natural biocontrol agents (Maina et al., 2017). Another avenue of research will explore the probiotic potential of LAB strains, as some bacteriocin-producing strains may also offer gut health benefits. Key parameters, such as their ability to adhere to intestinal cells, survive gastric conditions, and influence gut microbiota balance, will be assessed to determine their suitability as probiotic supplements (Anjana and Tiwari, 2022). If proven effective, these strains could have applications in both the food and pharmaceutical industries, particularly in functional foods or therapeutic probiotic formulations.

Before bacteriocins can be widely adopted, several industrial and regulatory challenges must be addressed. Large-scale production remains a hurdle due to high fermentation costs and potential yield limitations, necessitating optimization of growth conditions and biotechnological advancements to enhance production efficiency. Furthermore, bacteriocins must comply with food safety and pharmaceutical regulatory guidelines (e.g., EFSA, FDA), requiring rigorous toxicity, allergenicity, and resistance development assessments before approval (Liang et al., 2025; Garsa et al., 2014).

By integrating these aspects, future research will contribute to the development of bacteriocins as natural and sustainable antimicrobial agents. Their potential as antibiotic alternatives and biopreservatives aligns with increasing consumer demand for healthier, additive-free food products and novel antimicrobial solutions. Addressing the existing limitations through advanced molecular characterization, production optimization, stability testing, and clinical evaluation will pave the way for their successful application in food safety and medicine.

## Data Availability

The original contributions presented in the study are included in the article/supplementary material, further inquiries can be directed to the corresponding authors.
